# Comparative analysis of *Klebsiella pneumoniae* genomes identifies a phospholipase D family protein as a novel virulence factor

**DOI:** 10.1186/1741-7007-12-41

**Published:** 2014-05-29

**Authors:** Letícia MS Lery, Lionel Frangeul, Anna Tomas, Virginie Passet, Ana S Almeida, Suzanne Bialek-Davenet, Valérie Barbe, José A Bengoechea, Philippe Sansonetti, Sylvain Brisse, Régis Tournebize

**Affiliations:** 1 Institut Pasteur - Pathogénie Microbienne Moléculaire, Paris, France; 2 INSERM U786, Paris, France; 3 Institut Pasteur – Genome Bioinformatics Platform, Paris, France; 4 Laboratory Microbial Pathogenesis, Fundació d’Investigació Sanitària de les Illes Balears (FISIB), Bunyola, Spain; 5 Program Host-Pathogen Interactions, Centro de Investigación Biomédica en Red Enfermedades Respiratorias (CibeRes), Bunyola, Spain; 6 Institut Pasteur - Microbial Evolutionary Genomics, Paris, France; 7 CNRS, UMR3525, Paris, France; 8 CEA/IG/Genoscope, Finishing Laboratory, Evry, France; 9 Microbiologie et Maladies Infectieuses, Collège de France, Paris, France; 10 Present address: Biological Physics Laboratory, Institute of Biophysics Carlos Chagas Filho at the Federal University of Rio de Janeiro, Av. Carlos Chagas Filho, 373, Prédio do CCS, Bloco G, Sala G1-043, Rio de Janeiro, RJ 21941-902, Brazil; 11 Present address: Cold Spring Harbor Laboratory, Bungtown Road, Cold Spring Harbor, NY 11724, USA; 12 Present address: Centre for Infection and Immunity, Queen’s University Belfast, Belfast, UK; 13 Present address: Plate-Forme d’Imagerie Dynamique, Imagopole, Institut Pasteur, 28 rue du Docteur Roux, 75724 Paris, Cedex 15, France

**Keywords:** Genome sequencing, Host-microbe interactions, Bacterial pathogenesis, Lipid metabolism

## Abstract

**Background:**

*Klebsiella pneumoniae* strains are pathogenic to animals and humans, in which they are both a frequent cause of nosocomial infections and a re-emerging cause of severe community-acquired infections. *K. pneumoniae* isolates of the capsular serotype K2 are among the most virulent. In order to identify novel putative virulence factors that may account for the severity of K2 infections, the genome sequence of the K2 reference strain Kp52.145 was determined and compared to two K1 and K2 strains of low virulence and to the reference strains MGH 78578 and NTUH-K2044.

**Results:**

In addition to diverse functions related to host colonization and virulence encoded in genomic regions common to the four strains, four genomic islands specific for Kp52.145 were identified. These regions encoded genes for the synthesis of colibactin toxin, a putative cytotoxin outer membrane protein, secretion systems, nucleases and eukaryotic-like proteins. In addition, an insertion within a type VI secretion system locus included sel1 domain containing proteins and a phospholipase D family protein (PLD1). The *pld1* mutant was avirulent in a pneumonia model in mouse. The *pld1* mRNA was expressed *in vivo* and the *pld1* gene was associated with *K. pneumoniae* isolates from severe infections. Analysis of lipid composition of a defective *E. coli* strain complemented with *pld1* suggests an involvement of PLD1 in cardiolipin metabolism.

**Conclusions:**

Determination of the complete genome of the K2 reference strain identified several genomic islands comprising putative elements of pathogenicity. The role of PLD1 in pathogenesis was demonstrated for the first time and suggests that lipid metabolism is a novel virulence mechanism of *K. pneumoniae*.

## Background

*Klebsiella pneumoniae* is responsible for a variety of diseases in humans and animals. As a prominent nosocomial pathogen it is mainly responsible for urinary tract, respiratory tract or blood infections [1-3]. In addition, because of the acquisition of extended-spectrum β-lactamases and carbapenemases, such as the recently described NDM-1 [4], multi, extremely or pan-drug resistant clinical strains are more frequently isolated [5,6]. In addition, *K. pneumoniae* has re-emerged as a cause of community-acquired infections including pneumonia and the characteristic syndrome of pyogenic liver abscess, with possible complications including endophthalmitis or meningitis [7,8]. *K. pneumoniae* is, thus, an important virulent pathogen able to cause serious infections in ambulatory, otherwise healthy hosts and to spread within patients [5,9] that requires a better understanding of the molecular mechanisms underlying the various forms of its pathogenesis.

Major *K. pneumoniae* virulence factors include the capsule, the lipopolysaccharide, iron scavenging systems and adhesins [3,10-14]. The capsule is one of the most important virulence determinants, protecting against serum bactericidal activity, antimicrobial peptides and phagocytosis [11,15-18]. At least 77 capsular (K) types can be distinguished, but types K1 and K2 are prominent by their virulence in murine models of infection [19-21] and by their epidemiological prevalence [9,18,21,22]. However, not all K1/K2-type strains are necessarily virulent, as distinct clonal groups of K1 and K2 differ sharply by their virulence [23]. Reference strain Kp52.145 (derived from B5055, the reference strain of serotype K2) is a highly virulent strain from which important virulence factors, including the large virulence plasmid harboring the regulator of mucoid phenotype (*rmpA*) and the aerobactin cluster, were identified [10,11,21]. Even though a virulence plasmid-cured strain is less virulent than the parental strain, it remained more virulent than other isolates that do not harbor this plasmid [21], showing that factors other than capsule overexpression and aerobactin account for the higher pathogenesis of this strain. Therefore, although known virulence factors are certainly crucial for bacterial survival, protection and interaction with the host, putative new virulence factors that could subvert host cell physiology and response remain yet to be identified.

Comparative genomics of pathogenic and non-pathogenic strains is a powerful approach to identify putative virulence genes. Several draft or complete genomes of clinical isolates of *K. pneumoniae* have been published so far, but only the virulent serotype K1 strain NTUH-K2044 [24] has been described in detail. In order to identify new *K. pneumoniae* K2 virulence factors, we sequenced the genome of the virulent strain Kp52.145, as well as two additional strains of low virulence, SB2390 (serotype K2) and SB3193 (serotype K1). By comparing these three novel genomes to the publicly available genomes of the virulent K1 strain NTUH-K2044 and reference strain MGH 78578, we identified in Kp52.145 putative virulence genes and analyzed their distribution within a diverse collection of *K. pneumoniae* strains. We demonstrate that a gene coding for a phospholipase D family protein (PLD1), located within a type VI secretion system locus, is expressed *in vivo*, is involved in controlling bacterial membrane lipid composition, and is a new virulence factor.

## Results and Discussion

### Genome assembly and annotation

**Figure 1:**
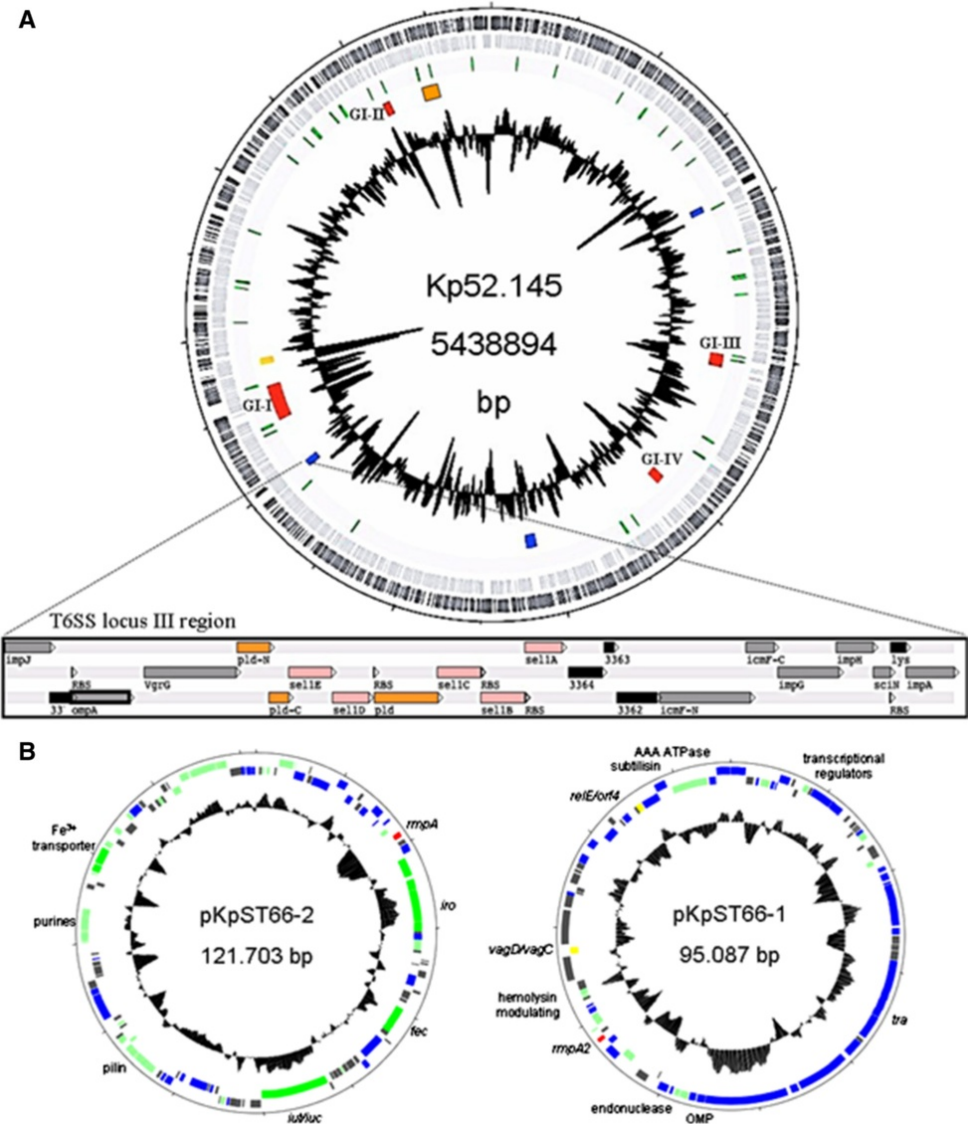
***K. pneumoniae *Kp52.145 genome. A)** Chromosome representation: Outermost layers in gray indicate the position of positive and negative strand CDSs. tRNAs are represented in green, while the four virulence-related genomic islands (GI) are in red, the locus coding for anaerobic metabolism in orange, the T6SS gene clusters in blue and capsular synthesis region in yellow. Inner circle represents G + C%. Detail of the T6SS locus III region containing the putative phospholipase (*pld1* gene) (orange) and *sel*1 (pink) genes is shown enlarged. **B)** Plasmids representation: plasmid maintenance genes and IS sequences are shown in blue, proteins with unknown functions in gray, known functions in green, toxin-antitoxin systems in yellow and *rmpA* in red. CDSs, coding sequences.

The genomes of Kp52.145, SB2390 and SB3193 were sequenced by a combination of 454 and Illumina technologies using single and paired-end libraries. Finishing efforts resulted in the complete genome sequence of *K. pneumoniae* Kp52.145 (one chromosome + two plasmids), comprising 5.45 Mbp and 5,314 protein coding genes (Figure 1). SB2390 and SB3193 genomes were assembled in 11 and 17 scaffolds, respectively. The GC% of these three genomes ranged from 55.6% to 56.7%. The general features of the *K. pneumoniae* sequenced genomes are summarized in Table 1.

**Table 1:** **General features of the *K. pneumoniae *** **genomes analyzed**

**K-type**	**Strain bank ID**	**Scaffolds**	**Size (Mb)**	**% GC**	**Total PCG**	**rRNA operons**	**tRNA genes**	**% coding**	**Plasmids**	**Reference**
K2	3341/Kp52.145	3	5.45	56.4	5,314	8	85	88	2	This manuscript
K2	2390	11	5.62	56.74	5,367	6	77	86.8	?	This manuscript
K1	3193	16	5.01	55.6	4,793	10	73	92	?	This manuscript
K1	NTUH (ST23)	2	5.24	57.6	4,992	19	76	89	1	Wu *et al*. [63]
K5	MGH (SB107)	6	5.31	57	4,776	10	56	85	5	(NC_009648)

The three genomes sequenced in this study are compared to strains NTUH and MGH, previously published. PCG, protein coding genes.

Because Kp52.145 is a highly virulent strain, our analyses were focused on comparing its genome with the genomes of *K. pneumoniae* strains SB2390, SB3193, NTUH-K2044 and MGH78578. According to SEED subsystems annotations [25], about 60% of protein-coding genes for each *K. pneumoniae* genome had predicted functions. More specifically, the largest percentage of annotated genes is involved in the metabolism of carbohydrates (approximately 20%), of amino acids and their derivatives (approximately 10%) and of cofactors, vitamins and prosthetic groups (approximately 8%) [see Additional file 1].

### Common genome

To define the common genome of the five strains, we used stringent BlastClust parameters of at least 90% identity and at least 80% coverage. This analysis identified 3,587 coding sequences common to the five genomes. The majority of proteins are involved in metabolic processes, such as energy metabolism and transporters, supporting the general concept that the core genome encompasses essential functions required for survival of the microorganism. The *K. pneumoniae* core genome comprised several sets of genes whose functions are related to bacterial survival in the environment or interaction with its host, and possibly virulence. This was the case, for example, of genes involved in quorum-sensing and biofilm formation, adhesins, and secretion systems, for which examples are detailed below.

Genes encoding for autoinducer-2 and type III fimbria, involved in biofilm formation in *K. pneumoniae*[26-28], were present in all sequenced strains. In addition, genes coding for synthesis and transport of the poly-β-1,6-N-acetyl-D-glucosamine (PGA) adhesin (KpST66_4915 to KpST66_4918 in Kp52.145 genome) which is required for the structural stability of *Escherichia coli* biofilms [29], and YidE (KpST66_0019), which mediates the hyperadherence phenotype of *E. coli*[30]*,* were also found in the core genome of *K. pneumoniae* strains.

Moreover, the five *K. pneumoniae* genomes contained the genes *bar*A/*uvr*Y (respectively KPST66_0986 and KpST66_3517) and *ycj*X/*ycj*F (KpST66_2441 and KpST66_2442) that may be involved in bacterial fitness and virulence. The two-component system BarA/UvrY (KpST66_3517 and KpST66_0986 in Kp52.145 genome) contributes to biofilm formation in *Salmonella enterica* and is a virulence determinant of urinary tract *E. coli* infections [31,32]. The *E. coli* YcjF protein is expressed in a septicemia murine model of infection in which the *ycj*F mutant is attenuated, thus suggesting its implication in the *in vivo* survival/multiplication of the bacteria [33].

Several putative secretion systems were identified as part of the common genome of the *K. pneumoniae* strains, including one type I secretion system (T1SS) and one type II secretion system (T2SS). T2SS is composed of the pullulanase related genes *pul*A-O that are involved in the pathogenesis of several bacteria [34,35]. *Streptococcus pyogenes* PulA binds to host lung glycogen leading to a strong interaction with alveolar type II cells [36]. Similarly, the alpha-amylase AmyA degrades glycogen into cyclic maltodextrins, which increases the transepithelial translocation of S*treptococcus*[37]. Both *amyA* and *pulA-O* genes are encoded in *K. pneumoniae* genomes, but their functions in this bacterium remain to be characterized. Type VI secretion system (T6SS) putative genes were located in at least three different loci of the *K. pneumoniae* genomes, in accordance with a previous *in-silico* study [38]. T6SS clusters are usually found within pathogenicity islands or on chromosomal regions presenting virulence or host survival biases. Additionally, T6SS has been suggested to assist colonization and infection. Indeed, in a screen that identified *K. pneumoniae* mutants failing to colonize mice [39], two of them were mutants in genes coding for proteins annotated as hypothetical, that have been subsequently re-annotated as T6SS proteins [38]. Type III secretion systems were not found in *Klebsiella*, but type IV secretion systems, possibly corresponding to conjugation apparatus, were present only in some strains (Kp52.145, SB2390 and NTUH-K2044) (Table 2).

**Table 2:** **Distribution of virulence-related factors among *K. pneumoniae *** **genomes analyzed**

**Virulence-factor**	**SB3341**	**SB2390**	**SB3193**	**NTUH**	**MGH**	**Functional role**
rmpA	++	-	-	++	-	Regulator of capsule expression
Aerobactin	+	-	-	+	-	Siderophore
Enterobactin	+	+	+	+	+	Siderophore
Yersiniabactin	+	+	+	+	-	Siderophore
Colibactin	+	-	-	-	-	Genotoxin
T4SS (virB)	+	+	-	+	-	Conjugative machinery/protein secretion
T2SS	+	+	+	+	+	Protein secretion
T6SS	+	+	+	+	+	Protein secretion
Pld-family	+	-	-	-	-	Lipid metabolism
Sel1 lipoproteins	+	-	-	-	-	Unknown
cOMP	+	-	-	-	-	Putative cytotoxin
Igg-like	+	-	-	-	-	Binding to extra cellular matrix compounds
SEFIR-domain	+	-	-	-	-	Potentially hijack IL17R signaling pathways
Bcl	+	+	-	+	+	Binding to hydrophobic ligands / putative regulation of homeostasis and immunity

Furthermore, the core genome presented a large genomic region located between gly-tRNA and phe-tRNA loci in Kp52.145 genome, containing *frdABCD* genes coding for the fumarate reductase enzymatic complex responsible for fumarate respiration under anaerobic growth of bacteria. This complex is a virulence determinant for *Helicobacter pylori*, *Mycobacterium tuberculosis*, *Actinobacillus pleuropneumoniae* and *S. enterica*, as mutants on these genes are attenuated [40-43]. The ability to grow anaerobically allows bacterial pathogens to persist in host tissues, including in the lungs. Curiously, this genomic locus encoded in *K. pneumoniae* at least one more protein involved in anaerobic metabolism, the anaerobic C4-dicarboxylate transporter DcuA (KpST66_4904), supporting the idea that this GI provides *K. pneumoniae* advantages to grow under anaerobic conditions, possibly favoring infection. Additional proteins that might be involved in bacterial fitness to environmental stress conditions were encoded in this region. For instance, the putative small multidrug resistance protein SugE (KpST66_4882) has been shown to regulate biofilm formation and capsule expression [44]. A lipocalin-2 bacterial protein Bcl (KpST66_4881) is also encoded in this island of the genome. Eukaryotic lipocalins are small extracellular proteins that bind hydrophobic ligands and fulfill numerous biological functions including regulation of cellular homeostasis and immunity and are regulators of antibacterial defense [45]. Lipocalin2 is for instance a siderophore scavenger for several bacteria, including *K. pneumoniae*[46], as well as a negative regulator of inflammatory response during *Streptococcus pneumoniae* pneumonia [47]. However, the role of bacterial lipocalins is not yet known.

Finally, a phosphatidylserine decarboxylase (KpST66_4873) might be important for the integrity of the bacterial membrane composition, as phospholipid biosynthetic pathways play crucial roles in the virulence of several pathogens [48,49].

### Accessory genome

The accessory genome (genes absent in at least one of the five strains) included a large number of specific coding sequences (CDSs): 743 genes were found only in Kp52.145, 608 in NTUH-K2044, 806 in SB3193, 635 in SB2390 and 488 in MGH78578. About 50% of these genes code for hypothetical proteins or proteins of unknown function. The distribution of the putative virulence-related genes of *K. pneumoniae* among the sequenced strains is summarized in Table 2. The specific regions or genes of Kp52.145 are detailed below.

### Kp52.145 plasmids

Strain Kp52.145 possessed two plasmids (Figure 1B). The first plasmid of 121 Kb carried the aerobactin cluster and the regulator of mucoid phenotype *rmpA* genes. The presence of this plasmid was previously correlated with the virulence of *K. pneumoniae* K1 and K2 isolates [21,50]. Additionally, a strain cured from this plasmid showed a 6 × 10^4^-fold reduction in virulence, establishing the link between this plasmid and bacterial virulence [21]. In addition to aerobactin and *rmpA*, this plasmid contained genes coding for F-pilin, purine metabolism, insertion sequences and proteins of unknown functions. Kp52.145 also carried a second, previously non-described, plasmid of about 90 Kb. Interestingly, it contained *rmpA2*, a homologue of the regulator of mucoid phenotype *rmpA*, which seems to be involved in capsule expression regulation [51,52]. F-pilin genes, a subtilisin-related serine protease, an AAA + ATPase, the UV protection system UmuD/UmuC and the genes encoding the toxin-antitoxin systems RelE/orf4 and VagD/VagC (Figure 1B) were also found on this plasmid. However, the potential role of these genes in virulence remains to be investigated.

### Genomic islands identified in the genome of strain Kp52.145

**Figure 2:**
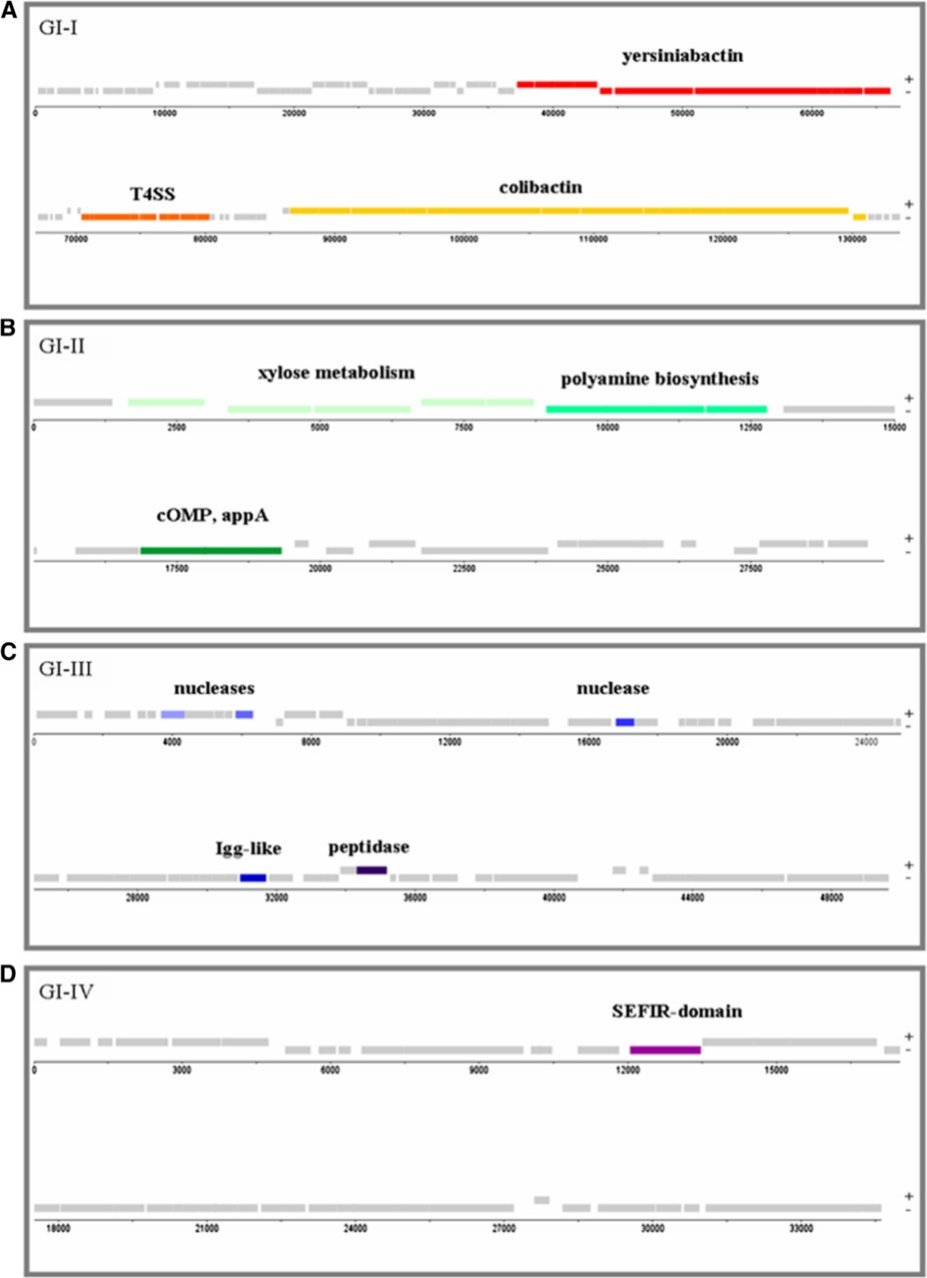
**Genomic islands (GIs) identified in the genome of Kp52.145.** Positive and negative strand CDSs are represented in gray. Putative virulence related genes are highlighted in different colors. The size of each GI is given in kb. Panel **A**: GI-I, **B**: GI-II, **C**: GI-III and **D**: GI-IV. CDS, coding sequences.

In addition to the capsule synthesis operon and the iron-acquisition systems (yellow and orange boxes in Figure 1A, respectively), known to be involved in *K. pneumoniae* virulence, four additional regions of the Kp52.145 genome presented several characteristics of pathogenicity islands (red boxes, Figure 1A). These regions are defined by a different GC content in comparison to the average of the genome, represented large chromosomal regions (often > 30 Kb), were associated with tRNA genes or with the presence of insertion sequences, integrases and transposases, and were present in pathogenic strains while less frequent in less-virulent strains [53,54]. The four GIs present in the Kp52.145 genome were present or partially present in NTUH, but none of them was present in SB2390, SB3193 and MGH 78578, indicating that their occurrence is specific to pathogenic genomes. Figure 2 shows the four GIs identified, highlighting the putative virulence related genes.

### Genomic island 1 (GI-1): ICE-Kp1-like region

The largest GI found in the Kp52.145 genome comprised 133,679 bp, presented a GC content of 52%, was inserted in an asn-tRNA locus and encoded 92 CDSs (Figure 2A). Most of the protein-coding genes found in this region were described as part of the IceKpI GI of NTUH-K2044 [55,56], although several differences were found. Kp52.145 GI-I begins at asn tRNA locus followed by several uncharacterized proteins and insertion sequence elements. GI-I coded for the synthesis of two polyketide/nonribosomal peptides (yersiniabactin and colibactin) and for the conjugative transfer machinery (T4SS) that allows horizontal transfer of the island [56]. In contrast to the previous description of IceKpI [56], the Kp52.145_GI-I carried colibactin and did not contain the region coding for *vagC-vagD*, *iroN-iroB-iroC-iroD* and *rmpA* genes which are carried only by the 121 kB plasmid in Kp52.145.

### Genomic island 2 (GI-II)

We describe here a novel GI (GI-II). It is a 29,829 bp island, with a GC content of 49% and coding for 28 CDSs which is inserted in a leu-tRNA locus (Figure 2B). Potential pathogenesis-related genes coded for a putative cytotoxic outer membrane protein (cOMP, KpST66_4736) and a polyamine ABC transport system (KpST66_4729 to KpST66_4732). cOMP closest known homologue (34% identity) was a *Plesiomonas shigelloides* predominant virulence factor proposed to trigger cell death in host cells following infection [57]. Polyamine biosynthesis and transport mechanisms were intricately linked to fitness, survival, biofilm formation and pathogenesis, for instance in *S. pneumoniae* and *Yersinia pestis*[58,59]. Additionally, this GI encoded a 4-phytase gene (KpST66_4736), *ugpQ3* (KpST66_4728) and *xylA*, *xynT*, *xynB*, *xylR* (KpST66_4724 to KpST66_4727) that are involved in xylose metabolism.

### Genomic island 3 (GI-III)

The third GI is characterized by a 49,657 bp region presenting a G + C content of 51% and contained 66 CDSs, most of them coding for phage structural proteins (Figure 2C). This GI included genes coding for proteins with homologues that were shown to play a role in bacterial adhesion and immune system escape [60-62]. These proteins encoded for an immunoglobulin domain-containing protein (KpST66_1506), a peptidase S24-like protein (KpST66_1511), two HNH family endonucleases (KpST66_1468 and 1486) and an exonuclease (locus 1464).

### Genomic island 4 (GI-IV)

The forth island (GI-IV) was mainly comprised of phage-related genes. Among the 42 CDSs encoded within this genomic region, one gene coded for a SEFIR-domain containing protein (KpST66_1945; Figure 2D). A SEFIR domain is usually found in IL17 receptors and SEF proteins, acting in eukaryotes signaling pathways. Very little is known about prokaryotic SEFIR-containing proteins. Structural analyses suggested that these bacterial SEFIR domains share structural and electrostatic similarity with their mammalian homologues and, thereby, could potentially subvert host immunity by hijacking the IL17R signaling pathways [63]. Notably, local production of IL-17 is a significant factor in effective host defense against Gram-negative bacteria, including *K. pneumoniae*[64]. Therefore, further studies are required to elucidate whether KpST66_1945 is implicated in *K. pneumoniae* pathogenesis.

### Distribution of GIs among *K. pneumoniae* strains

To investigate the distribution of the described GIs in *K. pneumoniae*, the presence of the putative virulence-related genes was searched using BlastN in 171 genomes, including 119 publicly available and 52 unpublished genomes (Bialek-Davenet, Brisse *et al*., unpublished work) representing 47 different sequence types (STs). Whereas the SEFIR-domain containing protein gene of GI-IV was only found in two (1.2%) isolates of sequence type ST15, the three other GIs described herein were more distributed among *K. pneumoniae* isolates (Table 3). GI-II genes were present in a total of 11 (6.4%) isolates, most of which belonged to ST375, ST65 and ST25, which were associated with severe infections caused by isolates of capsular serotype K2 [9,65]. The genes of GI-II were always found in synteny. GI-III genes were observed in only seven (4.1%) isolates dispersed in several unrelated STs. The distribution of the ICE*Kp*I element (similar to GI-I) has been previously analyzed [55].

**Table 3:** **Prevalence of the genomic islands among *K. pneumoniae *** **isolates**

**Genomic island**	**Virulence-related features**	**Prevalence (%)**	**Remarkable STs**
GI-I	Colibaction	3.5^a^	-
	Yersiniabactin		
GI-II	cOMP	5.8	3775,65,25
	4-phytase		
GI-III	Igg-like	8.1	Dispersed
	Exonuclease	57.6	375,65
GI-IV	Sefir-domain	1.7	-
T6SS insertion	PLD1	7.0	380,35
	Sel 1	7.0	380,35

^a^according to ref. [55]. Prevalence of virulence-related features encoded in GI-II, GI-III, GI-IV and T6SS insertion was based on >90% identity and >50% coverage in the length of the genes, using a database of 171 *Klebsiella* genomes representative of 47 different STs. STs, sequence types.

### T6SS locus III insertion

Recently, three different T6SS loci were defined in *K. pneumoniae*[38]. Within these loci, three putative valine-glycine repeat (Vgr) proteins and two hemolysin-coregulated proteins (Hcp) were described as potential effector proteins, through their sequence similarities to *Vibriocholerae* and *Pseudomonasaeruginosa* effector proteins [65-68]. Accordingly, the Kp52.145 genome also presented three conserved T6SS loci syntenic to those previously described. The first two loci were identical in composition and orientation to the previously described ones. The third one, locus III, had conservation of adjacency limited to the *imcF/impG/impH* and *impJ/ompA/vgrG* gene clusters, as a region with nine genes was inserted between these two clusters. This insertion encoded for one hypothetical protein, five putative Sel-1 repeat containing lipoproteins and three putative phospholipase D family proteins (Figure 1A). Flanking sequences suggesting how this region was inserted were not found.

**Figure 3:**
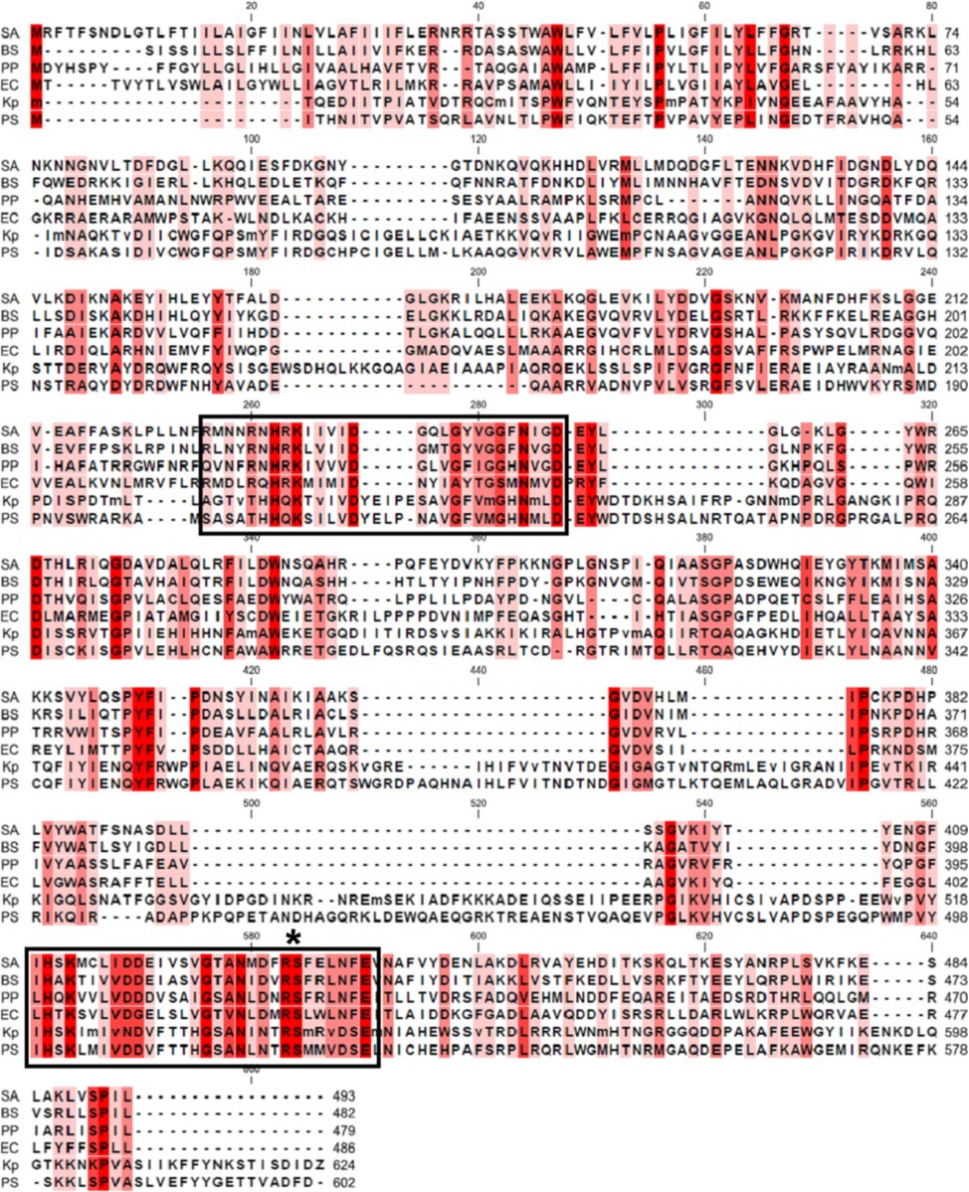
**Sequence alignment of Kp52.145 (Kp) PLD1 and its closest related sequences: putative phospholipase D family protein from *Pseudomonas syringae *(PS) and cardiolipin synthases from *P. putida (PP)*, *Staphylococcus aureus (SA), Bacilus subtilis (BS) *and *E. coli (EC)*.** The rectangles indicate the phospholipase D active site regions and the asterisk points to the corresponding transposon insertion site in *pld1* mutant strain.

Sel1 lipoproteins are poorly characterized and there was no evidence for their function in *K. pneumoniae*, but they are essential in *Legionellapneumophila* for invasion of host cells where they influence vacuolar trafficking of bacteria [69]. The three open reading frames encoding putative phospholipase D proteins in strain Kp52.145 encoded one full length protein (KpST66_3368, 623 aminoacids), one C-terminal region (KpST66_3371, 187 aa) and one N-terminal region (KpST66_3372, 317 aa). Phospholipase D family proteins have been described as important for host cell invasion, bacterial dissemination and disease progression [70-73]. The bacterial phospholipase D family comprises at least four classes of proteins with distinct functions: true phospholipase D, cardiolipin synthase, phosphatidylserine synthase and endonuclease [74]. Full length *K. pneumoniae* PLD1 and its closest homologs all presented the conserved motif HXK(X)_4_D and a serine or threonine approximately eight residues after asparagine (Figure 3), but no other conserved domain was described in each family, thus making it difficult to infer protein function only by sequence analysis.

In order to obtain evidence that the product of these three genes coding either for full length or partial PLD-family proteins are important for bacterial survival *in vivo*, we checked by RT-PCR for their mRNA expression. We observed that these genes are expressed both in bacteria grown for four hours in Trypto Casein Soy broth (GTCS) medium, as well as in the lungs of mice infected for 24 hours (data not shown). These results prompted us to further check for a putative involvement of the full length PLD gene, called *pld1*, on *K. pneumoniae* virulence.

### Involvement of the phospholipase D family protein gene *pld1* in KP 52.145 virulence

**Figure 4:**
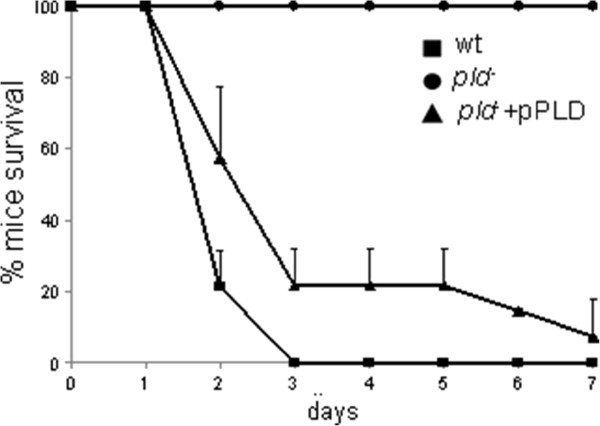
**PLD1 involvement in *K. pneumoniae *virulence.** Mice survival after infection with *K. pneumoniae* Kp52.145 wild-type, *pld1* mutant and complemented strains. Data are representative of seven mice per group from two independent experiments. Standard deviation is shown. PLD, phospholipase D.

As PLD-family proteins have been shown to be involved in virulence [75-77], we decided to characterize the role of the full length PLD1 protein. We first tested a *pld* mutant strain in a *K. pneumoniae* murine model. Mice were infected intranasally with 10^8^ of either the wild-type bacteria, a *pld1* mutant, or the mutant strain complemented with a plasmid expressing the putative PLD1 protein (pPLD), and their survival was monitored for seven days. Interestingly, the mutant strain appeared avirulent in a mouse model of acute pneumonia while mice infected with the wild-type and the complemented strain succumbed in less than one week (Figure 4). However, the wild-type, the mutant and the complemented mutant strains grew equally well in Luria-Bertani broth (LB) broth (data not shown). These results indicated that the *pld1* mutant was strongly attenuated *in vivo*, thus showing an important role for PLD1 in virulence.

To analyze the frequency and clonal distribution of the *pld1* gene in *K. pneumoniae*, the 171 genomes were analyzed using BlastN. We observed that besides ST66, represented by strain Kp52.145, *pld1* was present in 10 strains (6.4%) belonging to ST380, ST679, ST67 (*K. pneumoniae subsp. rhinoscleromatis*) and ST35, but in none of the other isolates. It is interesting to note that ST380 was associated with severe *K. pneumoniae* infections [9,65] and that *K. pneumoniae subsp. rhinoscleromatis* is the only *Klebsiella* strain to be able to survive intracellularly in macrophages [78].

### Functional characterization of PLD1

**Figure 5:**
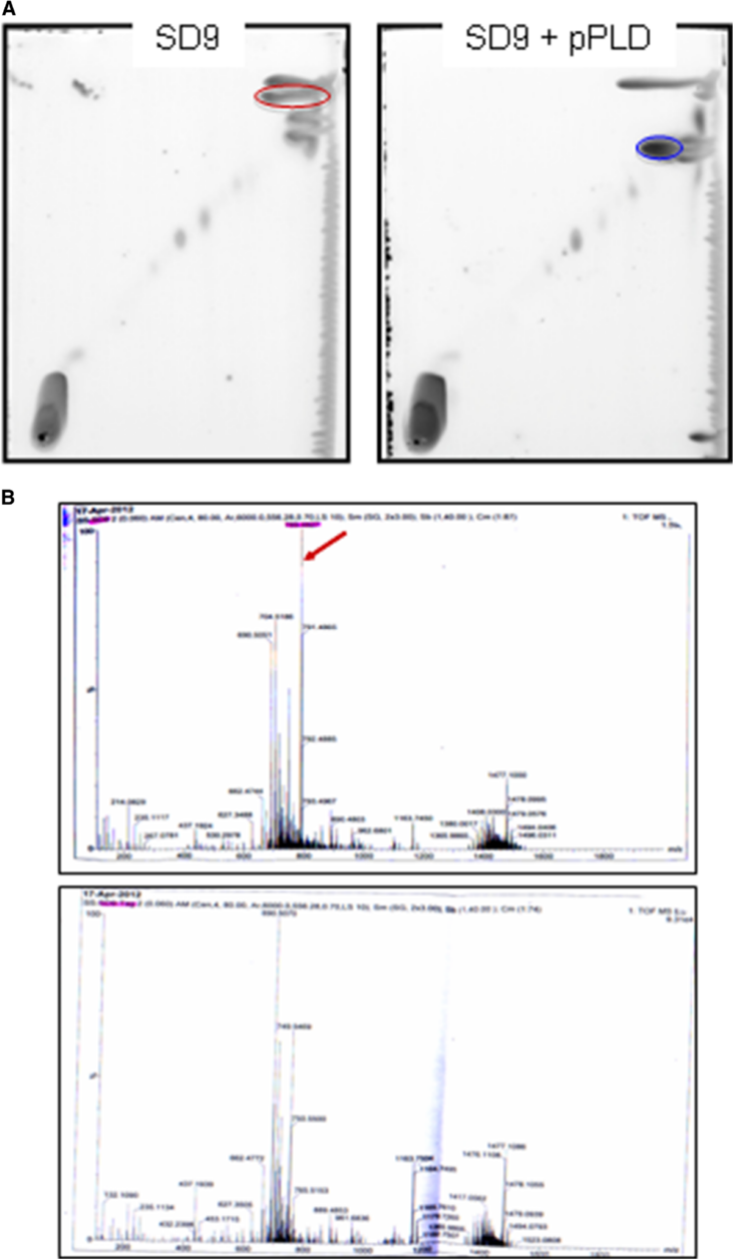
**PLD1 involvement in lipids metabolism. A)** TLC lipid profiles of *E. coli* strain SD9 and SD9 + pPLD. Red circle indicates phosphatidyl glycerol. Blue circle represents the lipid present specifically upon *pld1* expression. **B)** Mass spectrometry profiles of *E. coli* strain SD9 and SD9 + pPLD lipids. The red arrow points to m/z 789.4 exclusively found in wild-type strain. TLC, thin-layer chromatography.

In order to demonstrate the phospholipase activity of PLD1 and characterize its involvement in lipid metabolism, the lipid composition of wild-type and mutant strains was analyzed by thin-layer chromatography (TLC). A remarkable lipid spot was absent from the *pld1* mutant in comparison with the complemented strain, suggesting that the putative PLD1 is involved in lipid metabolism [see Additional file 1]. To reinforce this result, a plasmid carrying the *pld1* gene was inserted into *E. coli* strain SD9 [79]. This strain is deficient in phosphatidylserine and cardiolipin, thus presenting a simpler lipid composition than its parental strain and Kp52.145. Lipid profiles of SD9 and complemented strains had a different lipid composition. Notably, the PLD1-expressing strain contained an additional lipid spot in comparison to the SD9 strain, suggesting that PLD1 is responsible for this difference (Figure 5A). SD9 wild-type strain also presented an extra lipid spot in comparison to the PLD1*-*expressing strain, possibly representing the PLD1 substrate (Figure 5A). Densitometric analysis of iodine-stained lipids on TLC plates revealed that this lipid spot corresponded to 21% of the total amount of lipids in SD9 strain, but only 6% of SD9 complemented with a plasmid expressing *pld1*. Mass spectrometry (MS) analysis of total lipid extract was carried out to identify such lipid. Comparing lipid profiles by MS, we found a lipid of mass 788.4 present only in the PLD1*-*deficient strain (Figure 5B) and identified it as phosphatidyl glycerol (PG) using the LipidMaps database.

As mentioned above, the bacterial PLD-family proteins can be classified in four subfamilies. One of them, the cardiolipin synthase is able to convert two PG molecules into glycerol and cardiolipin, or to catalyze the opposite reaction, leading to PG formation [80]. Our results suggest that PLD1 belongs to the cardiolipin synthase subfamily and that it plays a role in balancing the PG and cardiolipin content.

It has been shown that humans and mice with bacterial pneumonia have markedly elevated amounts of cardiolipin in lung fluid and that it impairs surfactant function, lung mechanics, modulation of cell survival and cytokine networks and lung consolidation [81].

There is evidence that bacteria are able to adjust their relative concentrations of phosphatidylethanolamine and PGs when subjected to environmental stresses. Such an alteration in headgroup composition seems to be a means for changing membrane permeability and, hence, preserving stability [82]. Therefore, we hypothesize that PLD1 alters the membrane composition and charge, affecting bacterial interaction with the host environment.

Recently, Russel *el. al*. demonstrated that diverse phospholipase proteins encoded within the T6SS loci of several prokaryotic genomes are antibacterial effectors, conferring competitive advantages on the donor strain during interbacterial interactions [83]. These proteins are generally designated as ‘T6SS Lipase Effectors’ (Tle) and classified in five sub-families, according to the sequence conservation and number of catalytic motifs present. Kp52.145 PLD-family protein could be considered a Tle5 member, as it presents two conserved HxKxxxxD motifs. However, in the Kp52.145 genome we did not identify any gene similar to the cognate immunity genes - a hallmark of the genomic islands described by Russel *et al*. Moreover, we did not observe such an antibacterial effect of Kp52.145 or its PLD1 mutant strain upon competition with *E. coli* [see Additional file 1]. These results showed that *pld1* is implicated in virulence without being an anti-bacterial factor and is, so far, unique.

## Conclusions

This study presents a comparative analysis of the complete genome sequence of the high virulence reference strain Kp52.145, a derivative of the K2 reference strain B5055. It revealed five genomic regions possibly involved in bacterial virulence. One gene, *pld1*, was shown to be involved in virulence in a mouse model of pneumonia and revealed a novel implication of lipid metabolism in *K. pneumoniae* pathogenesis. Future analysis of additional putative virulence factors such as Sel1 lipoproteins, VgrG, Hcp, Bcl, cOMP and Sefir-domain containing protein are required for a comprehensive understanding of *K. pneumoniae* core virulence genes.

## Methods

### Selection of isolates for genome sequencing/bacterial strains

Strain Kp25.145 (a derivative of B5055, the reference strain of serotype K2) is a laboratory strain used to study *K. pneumoniae* pathogenesis [11,21] and was chosen as the focus of this work. SB2390 (cur15505, isolated in Curaçao, 2002, urinary tract infection; belongs to ST14) and SB3193 (IPEUC-744, isolated from a metritis case in a mare, 1981 in France; belonging to ST82) [23] are non-virulent strains that were sequenced to allow the comparison between virulent and non-virulent strains.

The *pld1* transposon mutant was isolated by Anna Tomas and Jose Bengoecheoa during a screen of a *K. pneumoniae* mutant library made by Tn5 transposon insertion (manuscript in preparation). In this mutant, the transposon was inserted at position 1,625 of *pld1* gene.

The complementation of the mutant strain was achieved through bacterial transformation using electrocompetent cells and a plasmid carrying *pld1* gene. *pld1* gene was amplified by PCR and cloned at the multiple cloning site of puc18 plasmid. Cloning was confirmed by DNA sequencing.

### Genome sequencing and assembly

*K. pneumoniae* strains were sequenced using a combination of 454 and Illumina reads. Single and paired-end 454 reads with an average of 400 nucleotides were assembled into contigs and scaffolds by Newbler. Illumina reads of about 76 or 36 nucleotides were aligned to scaffolds in order to confirm and correct possible homopolymer errors in the 454 reads. Coverage was as follows: Kp52.145 genome: 170 X using GAIIX (76 nt) + 13.8 X using MP titanium + 18 X using SR titanium; SB2390 genome: 81 X using GAIIX (36 nt) + 6.4 X using MP titanium + 22 X using SR titanium; SB3193 genome: 209 X using GAIIX (76 nt) + 5.4 X using MP titanium + 20 X using SR titanium.

Following the primary assembly of the genomes, an *in silico* finishing approach, based on the methods described by Pop *et al*. [84], was performed in order to identify small and identical repeats on the genomes. In such cases and without high quality bases discrepancies, the contigs containing the small repeats were manually duplicated and added to the assembly.

Scaffolds were aligned to experimentally determined *Bgl*II optical maps generated by OpGen company from purified chromosomal DNA, using MapSolver version 3.1 software. Such alignments were used to check for the quality of the assemblies. Additionally, specific pairs of primers were designed in order to close all remaining gaps. PCR products were purified in NucleoFast 96 plates (Macherey Nagel, Düren, Germany) and aliquots were used for sequencing reactions with the BigDye Terminator Cycle Sequencing Ready reaction Kit (Applied Biosystems, Foster City, CA, USA) on a ABI Prism 3730XL DNA Analyzer (Applied Biosystems). The resulting sequences were added to the previous assembly using Phred/Phrap/Consed.

The sequences of *K. pneumoniae* genomes have been deposited to the European Nucleotide Archive and are accessible under the accession numbers: FO834904, FO834905 and FO834906 (strain Kp52.145), CCBO00000000 (strain SB2390) and CCCQ000000000 (strain SB3193).

### Functional annotation of genomes

In order to gain functional insights about the genome sequences, protein-coding genes were predicted and annotated using the CAAT-box genome browser [85], using a combination of GeneMark predictions and Blastx results against the Uniprot database. All the putative open reading frames (ORF) longer than 120 nucleotides presenting similarity to sequences of the Uniprot database or positive GeneMark result were considered for further analysis. The final set of CDSs underwent a manual annotation process based on description of similarity. Pfam and COG database searches, as well as SignalP, TMHMM and PredTMBB predictions were performed to improve the degree of annotation confidence, if necessary. CDSs were described as ‘highly similar to’ , ‘similar to’ or ‘weakly similar to’ if they presented more than 70%, between 50% and 69% or less than 50% similarity to the protein hit sequence. Additionally, information on partial homology was included. The start codon for each CDS was automatically chosen and manually validated, based on a combination of GeneMark results and Blast alignments. RAST was used to classify proteins in functional categories. Structural RNAs were searched using tRNAscan.

The common genome of the five strains was determined using the BlastClust algorithm [86] using minimum parameters of 90% identity and 80% length coverage for proteins to be included in the same cluster.

### RT-PCR

Lungs of control or Kp52.145-infected mice were homogenized in cold TRI reagent (Sigma, Gillingham, Dorset, UK) using a Precellys lysing kit (Precellys, Saint Quentin en Yvelines, France). Total lung and bacterial mRNAs were extracted according to the manufacturer’s instructions. RNA (2 μg) was reversed transcribed in cDNA using Superscript II enzyme (Invitrogen, Foster City, CA, USA). Aliquots were used in PCR reactions using specific primers [see Additional file 1].

### Animal experiments

BALB/cJ mice were purchased from Janvier (Le Genest St. Isle, France). Mice were housed under standard conditions of feeding, light and temperature with access to food and water *ad libidium*. Experiments were performed according to the national and Institut Pasteur guidelines for laboratory animal experiments. Protocols were approved by the Institut Pasteur animal care and use committee (protocol 05–59) and the Direction des Services Vétérinaire de Paris (permit 75–713 to RT). Six- to eight-week-old mice were anesthetized with acepromazine (Calmivet, 1.5 mg/kg, Vetoquinol, Lure, France). and ketamine (Imalgene, 31.25 mg/kg, Merial, Lyon, France). and then infected intranasally with 20 μl bacterial suspension. The virulence of *K. pneumoniae* strains was tested on six-week-old BALB/c mice, as previously described [23]. Seven mice per test condition were infected with 10^8^ bacteria. Mice were followed every day for one week. Experiments were performed at least twice.

### Thin-layer chromatography and mass spectrometry of lipids

Bacterial lipids were extracted by the method of Bligh and Dyer [87]. Briefly, bacterial stationary-phase cultures were concentrated 10 times and mixed with chloroform:methanol. After centrifugation at 1,000 rpm for five minutes, the organic phase was recovered. Lipid profiles were analyzed by two-dimensional TLC using TLC Silica gel 60 F_254_ plates (Merck, Whitehouse Station, NJ, USA). as the stationary phase and a chloroform 9:1 methanol mixture as the mobile phase in both dimensions. Staining was performed by iodine vapor. TLC calibrated images were aquired in ImageScanner using LabScan v5.0 software. The relative intensity of each spot was calculated in ImageMaster two-dimensional Platinum v7.0 software. Alternatively, lipid extracts were analyzed by MS and MS/MS in an ESI-Q-Tof Micro (Waters), in positive ion mode. Resolution was typically lower than 10 ppm.

### Anti-bacterial competition assays

Competition assays were performed as previously described [88]. Briefly, *K. pneumoniae* Kp52.145 or its *pld1* mutant cells grown overnight on an agar plate were resuspended in LB, normalized to OD_600_ of 0.5 and mixed at a ratio of 5:1 with a spontaneous nalidixic acid (nal) resistant mutant strain of *E. coli* MG1655. The mixture was incubated for four hours on a prewarmed agar plate. Recovered cells were plated out on antibiotic selective media and viable cells were reported as the total number recovered per co-culture spot. *Serratia marcescens* DB10 was used as a positive control.

## Competing interests

The authors declare they have no competing interests.

## Authors’ contributions

LMSL participated in the genome sequencing and assembly, carried out the annotation and comparative genomic analysis, performed RT-PCR, lipid analysis and animal experiments and drafted the manuscript. LF carried out the genome assembly and participated in genome annotation. AT and JB generated the mutant strain and carried out the bacterial competition assay. VP, ASA and VB participated in the genome sequencing. SB participated in the design of the study and the genome sequencing and assembly. PS participated in the study design and coordination. RT conceived the study, participated in the genome sequencing and assembly and carried out animal experiments. SBD analyzed the presence of genes within other *K. pneumoniae* genomes. LMLS, JB, PJS, SB and RT wrote the manuscript. All authors read and approved the final manuscript.

## Supplementary Material

Additional file 1 **1) Distribution of *K. pneumoniae* Kp52.145 genes, according to RAST categories.** Each functional category is represented in a different color. The total number of genes per category is shown. 2) pld PCR assay. A collection of 42 virulent and non virulent clones was screened by PCR for *pld* gene. Strains containing *pld* gene are indicated by (+) and the absence of *pld* gene is (-). 3) TLC lipid profiles of *K. pneumoniae* Kp52.145 wild-type (left panel) and *pld* mutant strains (right panel). Black circles indicate differentially expressed lipids. 4) Bacterial competition assay. Anti-bacterial activity was measured as the number of *E. coli* cells recovered after the co-culture with *K. pneumoniae* Kp52.145 wild-type and *pld* mutant strains. *S. marcesens* was used as a positive control strain. 5) List of primers used for RT-PCR analysis. Click here for file

## References

[B1] Brisse S Grimont F Grimont PAD The genus Klebsiella The Prokaryotes: A Handbook on the Biology of Bacteria 2006 12 3 New York: Springer NY 159 196

[B2] Keynan Y Rubinstein E The changing face of Klebsiella pneumoniae infections in the community Int J Antimicrob Agents 2007 12 385 389 10.1016/j.ijantimicag.2007.06.019 17716872

[B3] Podschun R Ullmann U Klebsiella spp. as nosocomial pathogens: epidemiology, taxonomy, typing methods, and pathogenicity factors Clin Microbiol Rev 1998 12 589 603 9767057 10.1128/cmr.11.4.589 PMC88898

[B4] Moellering RC Jr NDM-1–a cause for worldwide concern N Engl J Med 2010 12 2377 2379 10.1056/NEJMp1011715 21158655

[B5] Shon AS Russo TA Hypervirulent Klebsiella pneumoniae: the next superbug? Future Microbiol 2012 12 669 671 10.2217/fmb.12.43 22702521

[B6] Su SC Siu LK Ma L Yeh KM Fung CP Lin JC Chang FY Community-acquired liver abscess caused by serotype K1 Klebsiella pneumoniae with CTX-M-15-type extended-spectrum beta-lactamase Antimicrob Agents Chemother 2008 12 804 805 10.1128/AAC.01269-07 18056273 PMC2224741

[B7] Siu LK Yeh KM Lin JC Fung CP Chang FY Klebsiella pneumoniae liver abscess: a new invasive syndrome Lancet Infect Dis 2012 12 881 887 10.1016/S1473-3099(12)70205-0 23099082

[B8] Yoon JH Kim YJ Jun YH Kim SI Kang JY Suk KT Kim DJ Liver abscess due to Klebsiella pneumoniae: Risk factors for metastatic infection Scand J Infect Dis 2014 12 21 26 10.3109/00365548.2013.851414 24228822

[B9] Decre D Verdet C Emirian A Le Gourrierec T Petit JC Offenstadt G Maury E Brisse S Arlet G Emerging severe and fatal infections due to Klebsiella pneumoniae in two university hospitals in France J Clin Microbiol 2011 12 3012 3014 10.1128/JCM.00676-11 21677064 PMC3147753

[B10] Nassif X Honore N Vasselon T Cole ST Sansonetti PJ Positive control of colanic acid synthesis in Escherichia coli by rmpA and rmpB, two virulence-plasmid genes of Klebsiella pneumoniae Mol Microbiol 1989 12 1349 1359 10.1111/j.1365-2958.1989.tb00116.x 2693894

[B11] Nassif X Fournier JM Arondel J Sansonetti PJ Mucoid phenotype of Klebsiella pneumoniae is a plasmid-encoded virulence factor Infect Immun 1989 12 546 552 2643575 10.1128/iai.57.2.546-552.1989 PMC313131

[B12] Regue M Hita B Pique N Izquierdo L Merino S Fresno S Benedi VJ Tomas JM A gene, uge, is essential for Klebsiella pneumoniae virulence Infect Immun 2004 12 54 61 10.1128/IAI.72.1.54-61.2004 14688080 PMC343961

[B13] Mey A Ponard D Colomb M Normier G Binz H Revillard JP Acylation of the lipid A region of a Klebsiella pneumoniae LPS controls the alternative pathway activation of human complement Mol Immunol 1994 12 1239 1246 10.1016/0161-5890(94)90074-4 7969185

[B14] Izquierdo L Coderch N Pique N Bedini E Corsaro MM Merino S Fresno S Tomas JM Regue M The Klebsiella pneumoniae wabG gene: role in biosynthesis of the core lipopolysaccharide and virulence J Bacteriol 2003 12 7213 7221 10.1128/JB.185.24.7213-7221.2003 14645282 PMC296265

[B15] Llobet E Tomas JM Bengoechea JA Capsule polysaccharide is a bacterial decoy for antimicrobial peptides Microbiology 2008 12 3877 3886 10.1099/mic.0.2008/022301-0 19047754

[B16] Campos MA Vargas MA Regueiro V Llompart CM Alberti S Bengoechea JA Capsule polysaccharide mediates bacterial resistance to antimicrobial peptides Infect Immun 2004 12 7107 7114 10.1128/IAI.72.12.7107-7114.2004 15557634 PMC529140

[B17] Moranta D Regueiro V March C Llobet E Margareto J Larrarte E Garmendia J Bengoechea JA Klebsiella pneumoniae capsule polysaccharide impedes the expression of beta-defensins by airway epithelial cells Infect Immun 2010 12 1135 1146 10.1128/IAI.00940-09 20008534 PMC2825953

[B18] Yeh KM Kurup A Siu LK Koh YL Fung CP Lin JC Chen TL Chang FY Koh TH Capsular serotype K1 or K2, rather than magA and rmpA, is a major virulence determinant for Klebsiella pneumoniae liver abscess in Singapore and Taiwan J Clin Microbiol 2007 12 466 471 10.1128/JCM.01150-06 17151209 PMC1829066

[B19] Mizuta K Ohta M Mori M Hasegawa T Nakashima I Kato N Virulence for mice of Klebsiella strains belonging to the O1 group: relationship to their capsular (K) types Infect Immun 1983 12 56 61 6187694 10.1128/iai.40.1.56-61.1983 PMC264817

[B20] Simoons-Smit AM Verwey-van Vught AM Kanis IY MacLaren DM Virulence of Klebsiella strains in experimentally induced skin lesions in the mouse J Med Microbiol 1984 12 67 77 10.1099/00222615-17-1-67 6363708

[B21] Nassif X Sansonetti PJ Correlation of the virulence of Klebsiella pneumoniae K1 and K2 with the presence of a plasmid encoding aerobactin Infect Immun 1986 12 603 608 2946641 10.1128/iai.54.3.603-608.1986 PMC260211

[B22] Fung CP Chang FY Lee SC Hu BS Kuo BI Liu CY Ho M Siu LK A global emerging disease of Klebsiella pneumoniae liver abscess: is serotype K1 an important factor for complicated endophthalmitis? Gut 2002 12 420 424 10.1136/gut.50.3.420 11839725 PMC1773126

[B23] Brisse S Fevre C Passet V Issenhuth-Jeanjean S Tournebize R Diancourt L Grimont P Virulent clones of Klebsiella pneumoniae: identification and evolutionary scenario based on genomic and phenotypic characterization PLoS One 2009 12 e4982 10.1371/journal.pone.0004982 19319196 PMC2656620

[B24] Wu KM Li LH Yan JJ Tsao N Liao TL Tsai HC Fung CP Chen HJ Liu YM Wang JT Fang CT Chang SC Shu HY Liu TT Chen YT Shiau YR Lauderdale TL Su IJ Kirby R Tsai SF Genome sequencing and comparative analysis of Klebsiella pneumoniae NTUH-K2044, a strain causing liver abscess and meningitis J Bacteriol 2009 12 4492 4501 10.1128/JB.00315-09 19447910 PMC2704730

[B25] Aziz RK Bartels D Best AA DeJongh M Disz T Edwards RA Formsma K Gerdes S Glass EM Kubal M Meyer F Olsen GJ Olson R Osterman AL Overbeek RA McNeil LK Paarmann D Paczian T Parrello B Pusch GD Reich C Stevens R Vassieva O Vonstein V Wilke A Zagnitko O The RAST Server: rapid annotations using subsystems technology BMC Genomics 2008 12 75 10.1186/1471-2164-9-75 18261238 PMC2265698

[B26] Di Martino P Cafferini N Joly B Darfeuille-Michaud A Klebsiella pneumoniae type 3 pili facilitate adherence and biofilm formation on abiotic surfaces Res Microbiol 2003 12 9 16 10.1016/S0923-2508(02)00004-9 12576153

[B27] Lavender HF Jagnow JR Clegg S Biofilm formation in vitro and virulence in vivo of mutants of Klebsiella pneumoniae Infect Immun 2004 12 4888 4890 10.1128/IAI.72.8.4888-4890.2004 15271955 PMC470696

[B28] Balestrino D Haagensen JA Rich C Forestier C Characterization of type 2 quorum sensing in Klebsiella pneumoniae and relationship with biofilm formation J Bacteriol 2005 12 2870 2880 10.1128/JB.187.8.2870-2880.2005 15805533 PMC1070389

[B29] Agladze K Wang X Romeo T Spatial periodicity of Escherichia coli K-12 biofilm microstructure initiates during a reversible, polar attachment phase of development and requires the polysaccharide adhesin PGA J Bacteriol 2005 12 8237 8246 10.1128/JB.187.24.8237-8246.2005 16321928 PMC1317006

[B30] Torres AG Jeter C Langley W Matthysse AG Differential binding of Escherichia coli O157:H7 to alfalfa, human epithelial cells, and plastic is mediated by a variety of surface structures Appl Environ Microbiol 2005 12 8008 8015 10.1128/AEM.71.12.8008-8015.2005 16332780 PMC1317338

[B31] Teplitski M Al-Agely A Ahmer BM Contribution of the SirA regulon to biofilm formation in Salmonella enterica serovar Typhimurium Microbiology 2006 12 3411 3424 10.1099/mic.0.29118-0 17074910

[B32] Suzuki K Wang X Weilbacher T Pernestig AK Melefors O Georgellis D Babitzke P Romeo T Regulatory circuitry of the CsrA/CsrB and BarA/UvrY systems of Escherichia coli J Bacteriol 2002 12 5130 5140 10.1128/JB.184.18.5130-5140.2002 12193630 PMC135316

[B33] Khan MA Isaacson RE Identification of Escherichia coli genes that are specifically expressed in a murine model of septicemic infection Infect Immun 2002 12 3404 3412 10.1128/IAI.70.7.3404-3412.2002 12065479 PMC128117

[B34] Baldi DL Higginson EE Hocking DM Praszkier J Cavaliere R James CE Bennett-Wood V Azzopardi KI Turnbull L Lithgow T Robins-Browne RM Whitchurch CB Tauschek M The Type II secretion system and its ubiquitous lipoprotein substrate, SslE, are required for biofilm formation and virulence of enteropathogenic Escherichia coli Infect Immun 2012 12 2042 2052 10.1128/IAI.06160-11 22451516 PMC3370571

[B35] Iwobi A Heesemann J Garcia E Igwe E Noelting C Rakin A Novel virulence-associated type II secretion system unique to high-pathogenicity Yersinia enterocolitica Infect Immun 2003 12 1872 1879 10.1128/IAI.71.4.1872-1879.2003 12654803 PMC152056

[B36] Hytonen J Haataja S Finne J Streptococcus pyogenes glycoprotein-binding strepadhesin activity is mediated by a surface-associated carbohydrate-degrading enzyme, pullulanase Infect Immun 2003 12 784 793 10.1128/IAI.71.2.784-793.2003 12540558 PMC145387

[B37] Shelburne SA 3rd Keith DB Davenport MT Beres SB Carroll RK Musser JM Contribution of AmyA, an extracellular alpha-glucan degrading enzyme, to group A streptococcal host-pathogen interaction Mol Microbiol 2009 12 159 174 10.1111/j.1365-2958.2009.06858.x 19735442 PMC4557622

[B38] Sarris PF Zoumadakis C Panopoulos NJ Scoulica EV Distribution of the putative type VI secretion system core genes in Klebsiella spp Infect Genet Evol 2011 12 157 166 10.1016/j.meegid.2010.09.006 20932940

[B39] Lawlor MS Hsu J Rick PD Miller VL Identification of Klebsiella pneumoniae virulence determinants using an intranasal infection model Mol Microbiol 2005 12 1054 1073 10.1111/j.1365-2958.2005.04918.x 16262790

[B40] Ge Z Feng Y Dangler CA Xu S Taylor NS Fox JG Fumarate reductase is essential for Helicobacter pylori colonization of the mouse stomach Microb Pathog 2000 12 279 287 10.1006/mpat.2000.0391 11031122

[B41] Li AH Lam WL Stokes RW Characterization of genes differentially expressed within macrophages by virulent and attenuated Mycobacterium tuberculosis identifies candidate genes involved in intracellular growth Microbiology 2008 12 2291 2303 10.1099/mic.0.2008/019661-0 18667562

[B42] Buettner FF Bendallah IM Bosse JT Dreckmann K Nash JH Langford PR Gerlach GF Analysis of the Actinobacillus pleuropneumoniae ArcA regulon identifies fumarate reductase as a determinant of virulence Infect Immun 2008 12 2284 2295 10.1128/IAI.01540-07 18378638 PMC2423083

[B43] Mercado-Lubo R Gauger EJ Leatham MP Conway T Cohen PS A Salmonella enterica serovar typhimurium succinate dehydrogenase/fumarate reductase double mutant is avirulent and immunogenic in BALB/c mice Infect Immun 2008 12 1128 1134 10.1128/IAI.01226-07 18086808 PMC2258826

[B44] Wu MC Lin TL Hsieh PF Yang HC Wang JT Isolation of genes involved in biofilm formation of a Klebsiella pneumoniae strain causing pyogenic liver abscess PLoS One 2011 12 e23500 10.1371/journal.pone.0023500 21858144 PMC3155550

[B45] Flower DR The lipocalin protein family: structure and function Biochem J 1996 12 1 14 8761444 10.1042/bj3180001 PMC1217580

[B46] Bachman MA Lenio S Schmidt L Oyler JE Weiser JN Interaction of lipocalin 2, transferrin, and siderophores determines the replicative niche of Klebsiella pneumoniae during pneumonia MBio 2012 12 6 10.1128/mBio.00224-11 PMC3509427 23169997

[B47] Warszawska JM Gawish R Sharif O Sigel S Doninger B Lakovits K Mesteri I Nairz M Boon L Spiel A Fuhrmann V Strobl B Muller M Schenk P Weiss G Knapp S Lipocalin 2 deactivates macrophages and worsens pneumococcal pneumonia outcomes J Clin Invest 2013 12 3363 3372 10.1172/JCI67911 23863624 PMC3726165

[B48] Chen YL Montedonico AE Kauffman S Dunlap JR Menn FM Reynolds TB Phosphatidylserine synthase and phosphatidylserine decarboxylase are essential for cell wall integrity and virulence in Candida albicans Mol Microbiol 2010 12 1112 1132 10.1111/j.1365-2958.2009.07018.x 20132453

[B49] Conde-Alvarez R Grillo MJ Salcedo SP de Miguel MJ Fugier E Gorvel JP Moriyon I Iriarte M Synthesis of phosphatidylcholine, a typical eukaryotic phospholipid, is necessary for full virulence of the intracellular bacterial parasite Brucella abortus Cell Microbiol 2006 12 1322 1335 10.1111/j.1462-5822.2006.00712.x 16882035

[B50] Tang HL Chiang MK Liou WJ Chen YT Peng HL Chiou CS Liu KS Lu MC Tung KC Lai YC Correlation between Klebsiella pneumoniae carrying pLVPK-derived loci and abscess formation Eur J Clin Microbiol Infect Dis 2010 12 689 698 10.1007/s10096-010-0915-1 20383552

[B51] Wacharotayankun R Arakawa Y Ohta M Tanaka K Akashi T Mori M Kato N Enhancement of extracapsular polysaccharide synthesis in Klebsiella pneumoniae by RmpA2, which shows homology to NtrC and FixJ Infect Immun 1993 12 3164 3174 8335346 10.1128/iai.61.8.3164-3174.1993 PMC280984

[B52] Hsu CR Lin TL Chen YC Chou HC Wang JT The role of Klebsiella pneumoniae rmpA in capsular polysaccharide synthesis and virulence revisited Microbiology 2011 12 3446 3457 10.1099/mic.0.050336-0 21964731

[B53] Hochhut B Dobrindt U Hacker J Pathogenicity islands and their role in bacterial virulence and survival Contrib Microbiol 2005 12 234 254 15496783 10.1159/000081698

[B54] Gal-Mor O Finlay BB Pathogenicity islands: a molecular toolbox for bacterial virulence Cell Microbiol 2006 12 1707 1719 10.1111/j.1462-5822.2006.00794.x 16939533

[B55] Putze J Hennequin C Nougayrede JP Zhang W Homburg S Karch H Bringer MA Fayolle C Carniel E Rabsch W Oelschlaeger TA Oswald E Forestier C Hacker J Dobrindt U Genetic structure and distribution of the colibactin genomic island among members of the family Enterobacteriaceae Infect Immun 2009 12 4696 4703 10.1128/IAI.00522-09 19720753 PMC2772509

[B56] Lin TL Lee CZ Hsieh PF Tsai SF Wang JT Characterization of integrative and conjugative element ICEKp1-associated genomic heterogeneity in a Klebsiella pneumoniae strain isolated from a primary liver abscess J Bacteriol 2008 12 515 526 10.1128/JB.01219-07 17981959 PMC2223707

[B57] Tsugawa H Ogawa A Takehara S Kimura M Okawa Y Primary structure and function of a cytotoxic outer-membrane protein (ComP) of Plesiomonas shigelloides FEMS Microbiol Lett 2008 12 10 16 10.1111/j.1574-6968.2007.01041.x 18318838

[B58] Shah P Nanduri B Swiatlo E Ma Y Pendarvis K Polyamine biosynthesis and transport mechanisms are crucial for fitness and pathogenesis of Streptococcus pneumoniae Microbiology 2011 12 504 515 10.1099/mic.0.042564-0 20966092

[B59] Wortham BW Oliveira MA Fetherston JD Perry RD Polyamines are required for the expression of key Hms proteins important for Yersinia pestis biofilm formation Environ Microbiol 2010 12 2034 2047 10.1111/j.1462-2920.2010.02219.x 20406298 PMC3039482

[B60] Choy HA Kelley MM Chen TL Moller AK Matsunaga J Haake DA Physiological osmotic induction of Leptospira interrogans adhesion: LigA and LigB bind extracellular matrix proteins and fibrinogen Infect Immun 2007 12 2441 2450 10.1128/IAI.01635-06 17296754 PMC1865782

[B61] Castiblanco-Valencia MM Fraga TR Silva LB Monaris D Abreu PA Strobel S Jozsi M Isaac L Barbosa AS Leptospiral immunoglobulin-like proteins interact with human complement regulators factor H, FHL-1, FHR-1, and C4BP J Infect Dis 2012 12 995 1004 10.1093/infdis/jir875 22291192

[B62] Beiter K Wartha F Albiger B Normark S Zychlinsky A Henriques-Normark B An endonuclease allows Streptococcus pneumoniae to escape from neutrophil extracellular traps Curr Biol 2006 12 401 407 10.1016/j.cub.2006.01.056 16488875

[B63] Wu B Gong J Liu L Li T Wei T Bai Z Evolution of prokaryotic homologues of the eukaryotic SEFIR protein domain Gene 2012 12 160 166 10.1016/j.gene.2011.10.033 22037611

[B64] Ye P Garvey PB Zhang P Nelson S Bagby G Summer WR Schwarzenberger P Shellito JE Kolls JK Interleukin-17 and lung host defense against Klebsiella pneumoniae infection Am J Respir Cell Mol Biol 2001 12 335 340 10.1165/ajrcmb.25.3.4424 11588011

[B65] Bialek-Davenet S Nicolas-Chanoine MH Decre D Brisse S Microbiological and clinical characteristics of bacteraemia caused by the hypermucoviscosity phenotype of Klebsiella pneumoniae in Korea Epidemiol Infect 2013 12 141 188 10.1017/S0950268812002051 PMC9152061 23006593

[B66] Miyata ST Kitaoka M Wieteska L Frech C Chen N Pukatzki S The Vibrio cholerae Type VI secretion system: evaluating its role in the human disease cholera Front Microbiol 2010 12 117 21607085 10.3389/fmicb.2010.00117 PMC3095397

[B67] Shrivastava S Mande SS Identification and functional characterization of gene components of Type VI secretion system in bacterial genomes PLoS One 2008 12 e2955 10.1371/journal.pone.0002955 18698408 PMC2492809

[B68] Boyer F Fichant G Berthod J Vandenbrouck Y Attree I Dissecting the bacterial type VI secretion system by a genome wide in silico analysis: what can be learned from available microbial genomic resources? BMC Genomics 2009 12 104 10.1186/1471-2164-10-104 19284603 PMC2660368

[B69] Newton HJ Sansom FM Dao J McAlister AD Sloan J Cianciotto NP Hartland EL Sel1 repeat protein LpnE is a Legionella pneumophila virulence determinant that influences vacuolar trafficking Infect Immun 2007 12 5575 5585 10.1128/IAI.00443-07 17893138 PMC2168337

[B70] McNamara PJ Bradley GA Songer JG Targeted mutagenesis of the phospholipase D gene results in decreased virulence of Corynebacterium pseudotuberculosis Mol Microbiol 1994 12 921 930 10.1111/j.1365-2958.1994.tb01080.x 7934899

[B71] Hinnebusch J Cherepanov P Du Y Rudolph A Dixon JD Schwan T Forsberg A Murine toxin of Yersinia pestis shows phospholipase D activity but is not required for virulence in mice Int J Med Microbiol 2000 12 483 487 10.1016/S1438-4221(00)80070-3 11111930

[B72] McKean SC Davies JK Moore RJ Probing the heat shock response of Corynebacterium pseudotuberculosis: the major virulence factor, phospholipase D, is downregulated at 43 degrees C Res Microbiol 2007 12 279 286 10.1016/j.resmic.2006.12.006 17320354

[B73] Mohan Das V Ballal M Proteinase and phospholipase activity as virulence factors in Candida species isolated from blood Rev Iberoam Micol 2008 12 208 210 10.1016/S1130-1406(08)70050-0 19071887

[B74] Ponting CP Kerr ID A novel family of phospholipase D homologues that includes phospholipid synthases and putative endonucleases: identification of duplicated repeats and potential active site residues Protein Sci 1996 12 914 922 8732763 10.1002/pro.5560050513 PMC2143407

[B75] McKean SC Davies JK Moore RJ Expression of phospholipase D, the major virulence factor of Corynebacterium pseudotuberculosis, is regulated by multiple environmental factors and plays a role in macrophage death Microbiology 2007 12 2203 2211 10.1099/mic.0.2007/005926-0 17600064

[B76] Kuhle K Flieger A Legionella phospholipases implicated in virulence Curr Top Microbiol Immunol 2013 12 175 209 23925490 10.1007/82_2013_348

[B77] Whitworth T Popov VL Yu XJ Walker DH Bouyer DH Expression of the Rickettsia prowazekii pld or tlyC gene in Salmonella enterica serovar Typhimurium mediates phagosomal escape Infect Immun 2005 12 6668 6673 10.1128/IAI.73.10.6668-6673.2005 16177343 PMC1230948

[B78] Fevre C Almeida AS Taront S Pedron T Huerre M Prevost MC Kieusseian A Cumano A Brisse S Sansonetti PJ Tournebize R A novel murine model of rhinoscleroma identifies Mikulicz cells, the disease signature, as IL-10 dependent derivatives of inflammatory monocytes EMBO Mol Med 2013 12 516 530 10.1002/emmm.201202023 23554169 PMC3628109

[B79] Shibuya I Miyazaki C Ohta A Alteration of phospholipid composition by combined defects in phosphatidylserine and cardiolipin synthases and physiological consequences in Escherichia coli J Bacteriol 1985 12 1086 1092 2982784 10.1128/jb.161.3.1086-1092.1985 PMC215011

[B80] Schlame M Cardiolipin synthesis for the assembly of bacterial and mitochondrial membranes J Lipid Res 2008 12 1607 1620 10.1194/jlr.R700018-JLR200 18077827 PMC2444000

[B81] Ray NB Durairaj L Chen BB McVerry BJ Ryan AJ Donahoe M Waltenbaugh AK O'Donnell CP Henderson FC Etscheidt CA McCoy DM Agassandian M Hayes-Rowan EC Coon TA Butler PL Gakhar L Mathur SN Sieren JC Tyurina YY Kagan VE McLennan G Mallampalli RK Dynamic regulation of cardiolipin by the lipid pump Atp8b1 determines the severity of lung injury in experimental pneumonia Nat Med 2010 12 1120 1127 10.1038/nm.2213 20852622 PMC4500192

[B82] Lopes SC Neves CS Eaton P Gameiro P Improved model systems for bacterial membranes from differing species: the importance of varying composition in PE/PG/cardiolipin ternary mixtures Mol Membr Biol 2012 12 207 217 10.3109/09687688.2012.700491 22830986

[B83] Russell AB Leroux M Hathazi K Agnello DM Ishikawa T Wiggins PA Wai SN Mougous JD Diverse type VI secretion phospholipases are functionally plastic antibacterial effectors Nature 2013 12 508 512 10.1038/nature12074 23552891 PMC3652678

[B84] Nagarajan N Cook C Di Bonaventura M Ge H Richards A Bishop-Lilly KA DeSalle R Read TD Pop M Finishing genomes with limited resources: lessons from an ensemble of microbial genomes BMC Genomics 2010 12 242 10.1186/1471-2164-11-242 20398345 PMC2864248

[B85] Frangeul L Glaser P Rusniok C Buchrieser C Duchaud E Dehoux P Kunst F CAAT-Box, Contigs-Assembly and Annotation Tool-Box for genome sequencing projects Bioinformatics 2004 12 790 797 10.1093/bioinformatics/btg490 14752000

[B86] Altschul SF Madden TL Schaffer AA Zhang J Zhang Z Miller W Lipman DJ Gapped BLAST and PSI-BLAST: a new generation of protein database search programs Nucleic Acids Res 1997 12 3389 3402 10.1093/nar/25.17.3389 9254694 PMC146917

[B87] Bligh EG Dyer WJ A rapid method of total lipid extraction and purification Can J Biochem Physiol 1959 12 911 917 10.1139/o59-099 13671378

[B88] Murdoch SL Trunk K English G Fritsch MJ Pourkarimi E Coulthurst SJ The opportunistic pathogen Serratia marcescens utilizes type VI secretion to target bacterial competitors J Bacteriol 2011 12 6057 6069 10.1128/JB.05671-11 21890705 PMC3194891

