# Supplementary material for: Comparative analysis of Klebsiella pneumoniae genomes identifies a phospholipase D family protein as a novel virulence factor
Source: BMC Biol. 2014 May 29;12:41. doi: 10.1186/1741-7007-12-41 (PMC4068068; doi:10.1186/1741-7007-12-41)
Supplement: Additional file 1 — 1) Distribution of K. pneumoniae Kp52.145 genes, according to RAST categories. Each functional category is represented in a different color. The total number of genes per category is shown. 2) pld PCR assay. A collection of 42 virulent and non virulent clones was screened by PCR for pld gene. Strains containing pld gene are indicated by (+) and the absence of pld gene is (-). 3) TLC lipid profiles of K. pneumoniae Kp52.145 wild-type (left panel) and pld mutant strains (right panel). Black circles indicate differentially expressed lipids. 4) Bacterial competition assay. Anti-bacterial activity was measured as the number of E. coli cells recovered after the co-culture with K. pneumoniae Kp52.145 wild-type and pld mutant strains. S. marcesens was used as a positive control strain. 5) List of primers used for RT-PCR analysis. [file 1741-7007-12-41-S1.pdf]

Supplementary Material

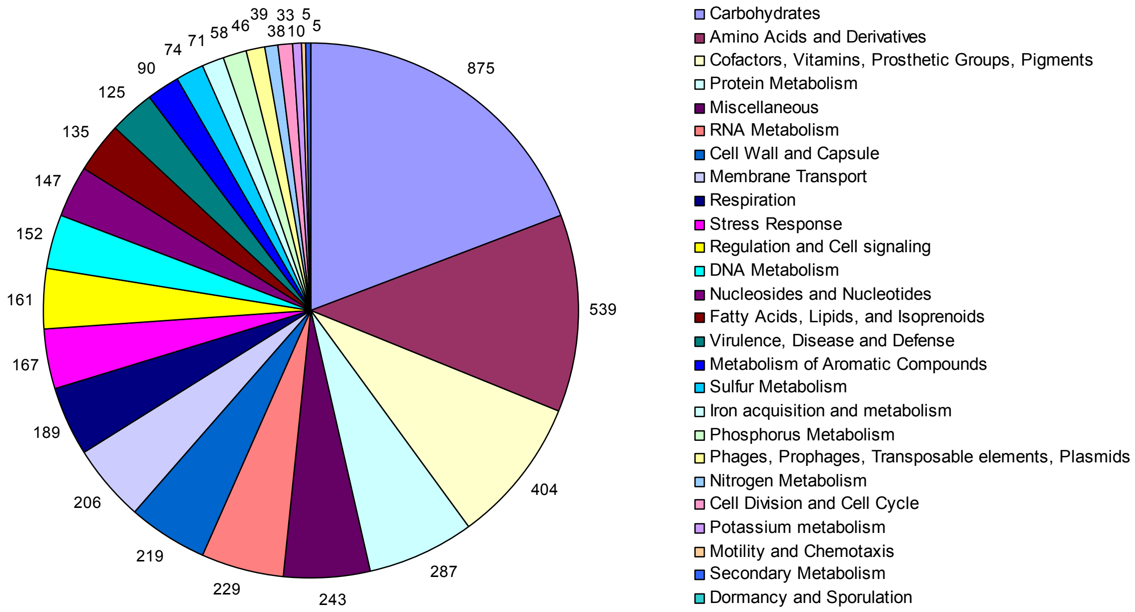

Sup. 1: Distribution of *K. pneumoniae* Kp52.145 genes, according to RAST categories. Each functional category is represented in a different color. The total number os genes per category is shown.

ST	PLD PCR	Infection
335	-	less virulent (nosocomial)
65	-	less virulent (nosocomial)
45	-	less virulent (nosocomial)
42	-	less virulent (nosocomial)
41	-	less virulent (nosocomial)
40	-	less virulent (nosocomial)
39	+	less virulent (nosocomial)
34	-	less virulent (nosocomial)
33	-	less virulent (nosocomial)
31	+	less virulent (nosocomial)
29	-	less virulent (nosocomial)
26	-	less virulent (nosocomial)
25	-	less virulent (nosocomial)
24	+	less virulent (nosocomial)
22	-	less virulent (nosocomial)
21	-	less virulent (nosocomial)
17	-	less virulent (nosocomial)
15	-	less virulent (nosocomial)
10	+	less virulent (nosocomial)
7	-	less virulent (nosocomial)
6	-	less virulent (nosocomial)
5	+	less virulent (nosocomial)

ST	PLD PCR	Infection
680	+	Virulent (Community-acquired and/or invasive)
679	+	Virulent (Community-acquired and/or invasive)
382	+	Virulent (Community-acquired and/or invasive)
380	+	Virulent (Community-acquired and/or invasive)
380	+	Virulent (Community-acquired and/or invasive)
380	+	Virulent (Community-acquired and/or invasive)
380	+	Virulent (Community-acquired and/or invasive)
380	+	Virulent (Community-acquired and/or invasive)
375	+	Virulent (Community-acquired and/or invasive)
375	-	Virulent (Community-acquired and/or invasive)
86	+	Virulent (Community-acquired and/or invasive)
86	+	Virulent (Community-acquired and/or invasive)
86	+	Virulent (Community-acquired and/or invasive)
86	+	Virulent (Community-acquired and/or invasive)
66	+	Virulent (Community-acquired and/or invasive)
65	+	Virulent (Community-acquired and/or invasive)
65	-	Virulent (Community-acquired and/or invasive)
57	+	Virulent (Community-acquired and/or invasive)
23	-	Virulent (Community-acquired and/or invasive)
23	-	Virulent (Community-acquired and/or invasive)
23	-	Virulent (Community-acquired and/or invasive)

Sup. 2: *pld* PCR assay. A collection of 42 virulent and non virulent clones was screened by PCR for *pld* gene. Strains containing pld gene are indicated by (+) and the absence of pld gene is (-).

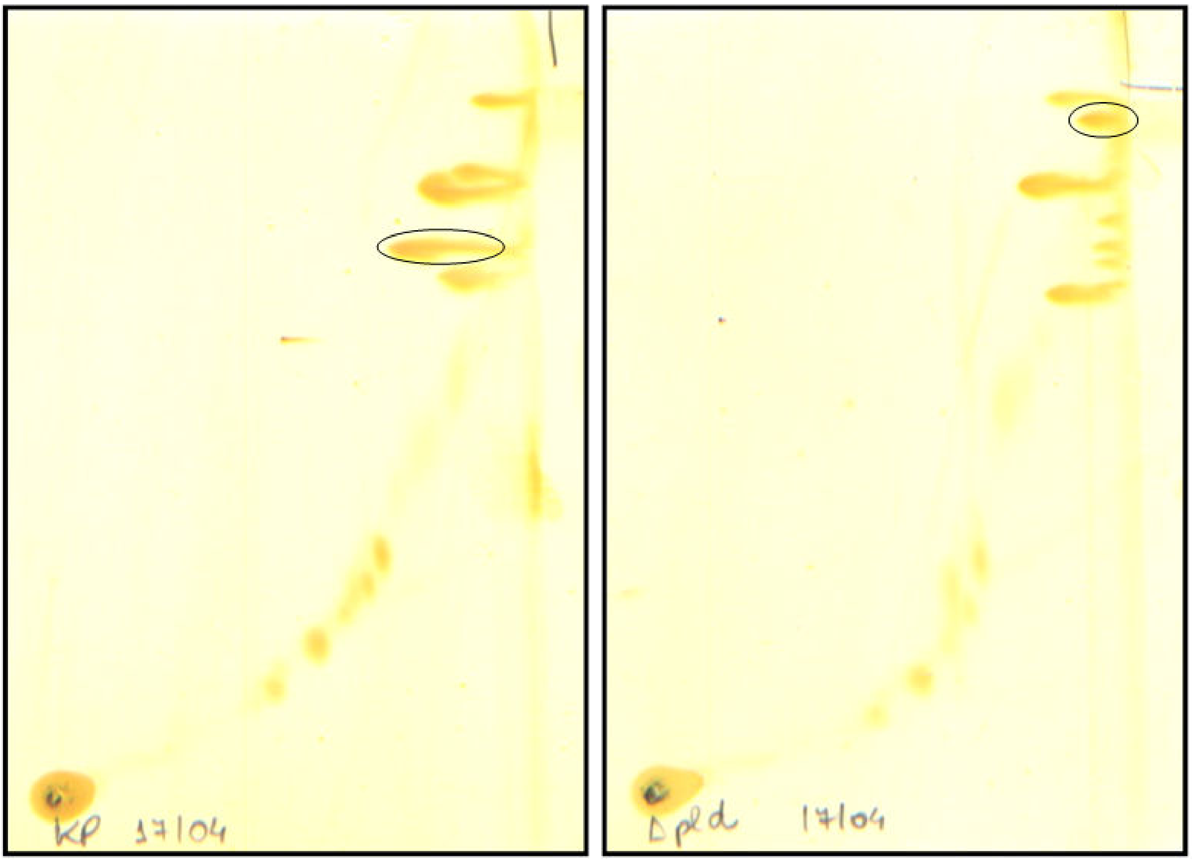

Sup. 3: TLC lipid profiles of *K. pneumoniae* Kp52.145 wild-type (left panel) and *pld* mutant strains (right panel). Black circles indicates differentially expressed lipids.

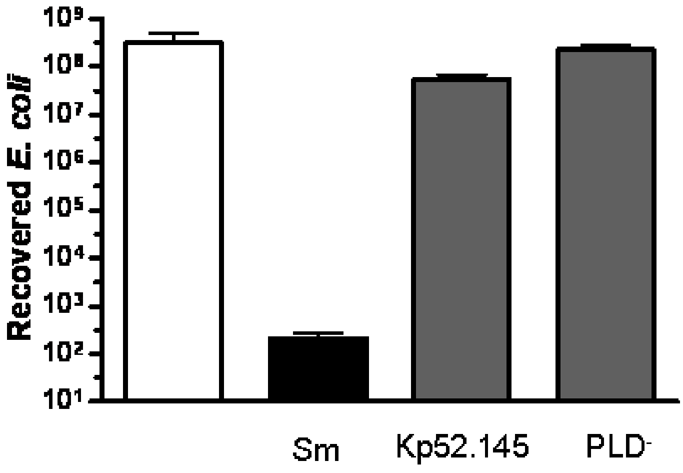

Sup. 4: Bacterial competition assay. Anti-bacterial activity was measured as the number of *E. coli* cells recovered after the co-culture with *K. pneumoniae* Kp52.145 wild-type and *pld* mutant strains. *S. marcesens* was used as a positive control strain.

Primer Name	Primer Sequence	Primer Name	Primer Sequence	Target Gene
6472F	CGTAATCGCGAGATGAGTGAG	6472R	CACCAGCGAGCAGATATGAAC	KpST66_3368
6477F	GCCGGCAGAAAGAATATGAAG	6477R	GCACTGAGCCCTTTATCGAGAG	KpST66_3371
6478F	CGTAATCGCGAGATGAGTGAG	6478R	CACCAGCGAGCAGATATGAAC	KpST66_3372
6476F	GGTGTGGCGGCTGGAGACAC	6476R	CGGGCACGTTCCGGATCCTT	KpST66_3370
6474F	AGCAGGGAGAGGGGAAGGCG	6474R	CCCGCAGCGACTCCCAACTG	KpST66_3369
6471F	TGCGTCGCTGTGCTGCTGAA	6471R	CGCTGCCACACCCAACTGGA	KpST66_3367
6465F	TGTATCGCATCGCGGCGGAA	6465R	CTCAGCGAAAAACGCCCGCG	KpST66_3366
6463F	CAGCGCCTGATGCAGTGCTG	6463R	CCGCCACGCTGTCCTTCTCA	KpST66_3365
79F	TCGATAACGCTTGGGCGATGGC	79R	TGTACCCGCTTTTGACGTTGGC	KpST66_4736

Sup. 5: List of primers used for RT-PCR analysis.
